# Phantom and clinical evaluation of the effect of full Monte Carlo collimator modelling in post-SIRT yttrium-90 Bremsstrahlung SPECT imaging

**DOI:** 10.1186/s13550-018-0361-0

**Published:** 2018-01-22

**Authors:** Charlotte A. Porter, Kevin M. Bradley, Eero T. Hippeläinen, Matthew D. Walker, Daniel R. McGowan

**Affiliations:** 1 Radiation Physics and Protection, Churchill Hospital, Oxford University Hospitals NHS Foundation Trust, Oxford, OX3 7LE UK; 2 Department of Radiology, Churchill Hospital, Oxford University Hospitals NHS Foundation Trust, Oxford, UK; 3 HUS Medical Imaging Centre, Clinical Physiology and Nuclear Medicine, University of Helsinki and Helsinki University Hospital, Helsinki, Finland; 4 Department of Oncology, University of Oxford, Old Road Campus Research Building, Oxford, UK

**Keywords:** Quantitative SPECT, Yttrium-90 Bremsstrahlung, Image reconstruction, Monte Carlo scatter correction

## Abstract

**Background:**

Post-therapy SPECT/CT imaging of ^90^Y microspheres delivered to hepatic malignancies is difficult, owing to the continuous, high-energy Bremsstrahlung spectrum emitted by ^90^Y. This study aimed to evaluate the utility of a commercially available software package (HybridRecon, Hermes Medical Solutions AB) which incorporates full Monte Carlo collimator modelling. Analysis of image quality was performed on both phantom and clinical images in order to ultimately provide a recommendation of an optimum reconstruction for post-therapy ^90^Y microsphere SPECT/CT imaging.

A 3D-printed anthropomorphic liver phantom was filled with ^90^Y with a sphere-to-background ratio of 4:1 and imaged on a GE Discovery 670 SPECT/CT camera. Datasets were reconstructed using ordered-subsets expectation maximization (OSEM) 1–7 iterations in order to identify the optimal OSEM reconstruction (5 iterations, 15 subsets). Quantitative analysis was subsequently carried out on phantom datasets obtained using four reconstruction algorithms: the default OSEM protocol (2 iterations, 10 subsets) and the optimised OSEM protocol, both with and without full Monte Carlo collimator modelling. The quantitative metrics contrast recovery (CR) and background variability (BV) were calculated.

The four algorithms were then used to retrospectively reconstruct 10 selective internal radiation therapy (SIRT) patient datasets which were subsequently blind scored for image quality by a consultant radiologist.

**Results:**

The optimised OSEM reconstruction (5 iterations, 15 subsets with full MC collimator modelling) increased the CR by 42% (*p* < 0.001) compared to the default OSEM protocol (2 iterations, 10 subsets). The use of full Monte Carlo collimator modelling was shown to further improve CR by 14% (30 mm sphere, CR = 90%, *p* < 0.05).

The consultant radiologist had a significant preference for the optimised OSEM over the default OSEM protocol (*p* < 0.001), with the optimised OSEM being the favoured reconstruction in every one of the 10 clinical cases presented.

**Conclusions:**

OSEM (5 iterations, 15 subsets) with full Monte Carlo collimator modelling is quantitatively the optimal image reconstruction for post-SIRT ^90^Y Bremsstrahlung SPECT/CT imaging. The use of full Monte Carlo collimator modelling for correction of image-degrading effects significantly increases contrast recovery without degrading clinical image quality.

## Background

Selective internal radiation therapy (SIRT) of hepatic malignancies involves delivery of ^90^Y microspheres to tumour via hepatic arteries [1]. Prior to administration of the ^90^Y microspheres, it is recommended that a ^99m^Tc MAA scan is performed to evaluate the shunting of activity to the lungs [1] but there is a debate about whether the MAA distribution reflects that of the therapeutic ^90^Y [2–4]. A post-therapy Bremsstrahlung scan was recommended in the 2007 report from the Radioembolization Brachytherapy Oncology Consortium [1], but more recent studies have demonstrated that PET may provide better quantification of ^90^Y distribution [5, 6]. As gamma cameras are cheaper and more numerous than PET scanners [7], their use for post-therapy imaging of ^90^Y SIRT is expected to continue. The improvement of ^90^Y Bremsstrahlung imaging will hence be beneficial to both current and future clinical needs.

Difficulties in imaging ^90^Y using SPECT arise from its continuous Bremsstrahlung spectrum with energies up to 2.3 MeV, meaning that traditional energy-based scatter rejection methods are ineffective [2]. The presence of scattered photons in SPECT images leads to degradations in contrast and thus in quantification [8].

Early methods of scatter reduction included the dual or triple energy window [9, 10] techniques. More modern, computationally heavy methods use Monte Carlo algorithms to model scatter in the object [11, 12] and, more recently, in the collimator and detector [13, 14].

Monte Carlo algorithms which model the scatter in the object are effective for radionuclides which have a single low-energy photopeak (e.g., ^99m^Tc) [13], when the detector response is assumed to be Gaussian [11], but for higher energy isotopes which do not have one single photopeak (e.g., ^90^Y), they are not effective. The full Monte Carlo (MC) collimator simulation in HybridRecon (HERMES Medical Solutions AB, Stockholm, Sweden) incorporates all photon interactions at the collimator, including both primary and scattered photons, in addition to considering scatter within the object. This is necessary owing to the increased amount of septal penetration, scatter within the collimator itself, and lead X-ray fluorescence with a higher energy radionuclide [13].

HybridRecon uses a scatter modelling method known as convolution-based forced detection [15]. The software uses pre-calculated probability density functions of the ^90^Y spectrum, which were generated using PENELOPE MC code [16], carries out the simulation according to Rault et al. [17], and utilises a β particle spectrum adopted from the Brookhaven National Laboratory database [18]. During the reconstruction, the photon energy is sampled from the Bremsstrahlung probability density function and the photon path at the object is modelled using the MC simulator. Scattered photons are forced to scatter towards the detector, producing 3D sub-projections; due to the continuous nature of the ^90^Y Bremsstrahlung spectrum, several sub-projections are created with different energy windows. Once the scatter sub-projections have been simulated, they are convolved to the detector plane with attenuation modelling. This convolution is carried out using pre-calculated MC depth-dependent Gaussian point spread functions which model the collimator and detector response.

The purpose of this work is firstly to optimise ordered-subsets expectation maximization (OSEM) reconstruction of ^90^Y SPECT and further to evaluate the effect of the advanced MC scatter correction offered by HybridRecon on image quality and quantification.

## Methods

### Phantom acquisitions

The AbdoMan™ phantom [19], a 3D-printed liver phantom with fillable spherical inserts, was used to perform quantitative evaluations of the reconstruction algorithms under test. The anthropomorphic nature of the AbdoMan™ phantom enabled quantitative analysis of image quality to be performed in a more clinically realistic scenario than using most commercially available phantoms.

Hot spheres of 10, 20, 30, and 40 mm diameter and a 40-mm hot sphere with a 25-mm solid (cold) centre were inserted into the warm liver background region with a sphere:background ratio of 4:1 and a clinically relevant total activity of 1340 MBq ^90^Y. The phantom was scanned on a GE Discovery 670 dual-headed SPECT/CT camera three times (GE Healthcare, Haifa, Israel) using the clinical protocol (energy window 50–150 keV, MEGP collimators, 128 × 128 matrix size, 360° acquisition, 90 views, 20 s/view) in order to obtain three acquisitions of the phantom.

**Fig. 1:**
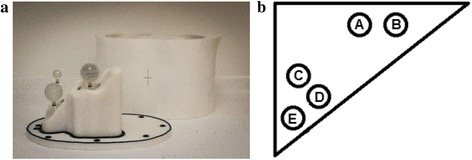
**a** The AbdoMan™ phantom, showing the fillable spheres mounted in the liver insert [19]*.* **b** Schematic representation of transaxial slice of AbdoMan™ phantom showing positions of spheres (A = 20-mm sphere, B = 40-mm sphere with 25-mm solid core, C = 40-mm sphere, D = 10-mm sphere, E = 30-mm sphere) N.B. Spheres were not all located on the same slice, but are shown here on the same slice for ease

The AbdoMan™ phantom is shown in Fig. 1 along with a schematic diagram of the position of the spheres within the phantom.

### Image reconstructions

HybridRecon (Version 1.1C, HERMES Medical Solutions AB) was used to perform all reconstructions whilst HybridViewer (Version 2.6F, HERMES Medical Solutions AB) was used to view and analyse the reconstructed images. All images were attenuation corrected using CT data and had the resolution recovery option switched on. Where full MC collimator modelling was not enabled, standard MC scatter correction was carried out.

One acquisition of the phantom was reconstructed with OSEM 1–7 iterations and 15 subsets in order to ascertain the optimum OSEM reconstruction. Previous work on optimisation of ^99m^Tc reconstruction in SPECT imaging had shown that five iterations and 16 subsets yield the best compromise between resolution and noise [20] but owing to the number of projections at which data were acquired at this institution, 16 subsets were not a possible option. Instead, the nearest alternative of 15 subsets was used. The optimum number of OSEM iterations for ^90^Y was found via quantitative analysis as described below.

Once the optimised OSEM reconstruction had been identified, all three of the acquisitions were reconstructed with the GE default OSEM protocol (2 iterations and 10 subsets) and the optimised OSEM protocol, both with and without full MC collimator modelling.

### Quantitative phantom analysis

Quantitative analysis on phantom datasets was carried out on unfiltered images. Circular regions of interest (ROIs) were drawn onto the hot spheres, on the widest slice of the sphere. ROIs of diameters equal to those of the hot spheres were drawn in the background region, positioned concentrically on three slices: one near the top, the bottom, and the middle of the phantom. The regions were drawn onto transaxial slices of the CT image and copied across to the aligned SPECT images.

Contrast recovery (CR) and background variability (BV) were both calculated as in the NEMA standard [21]. Data were exported into Microsoft Excel (2010) for analysis, with statistical analysis being carried out using IBM SPSS Statistics (Version 22.0, 2013).

### Clinical evaluation

Having optimised the reconstructions using phantom datasets, the raw datasets of 10 consecutive patients (administered activity range = 860–2370 MBq; mean = 1570 MBq) who had undergone SIRT therapy for metastatic colorectal cancer were retrospectively reconstructed, a practice which does not require informed consent at this institution. As with the phantom datasets, each patient dataset was reconstructed using four different methods: the default OSEM protocol and the optimised OSEM protocol, both with and without full MC collimator modelling. For the clinical evaluation, a 0.4-cm^−1^ Butterworth filter was applied to the images using HybridRecon.

A consultant radiologist, blinded to the reconstruction method, scored the images according to the criteria in Table 1. The radiologist was further asked to comment upon the visibility of any extrahepatic uptake, if present, and to state their preferred reconstruction for each patient. Access to prior diagnostic contrast-enhanced computed tomography and magnetic resonance imaging, including diffusion weighted images, of the liver were also available for each patient, to provide information regarding the location of the metastases and the expected texture of the liver.

**Table 1:** Criteria against which the consultant radiologist was asked to score the patient reconstructions

Category	Possible scores
Overall image quality	1, poor—5, excellent
Lesion detectability	1, poor—5, excellent
Visibility of necrotic regions (where applicable)	1, poor—5, excellent
Liver background noise	1, unacceptable—5, minimal

## Results

### Phantom acquisitions

Since the CR of the 10- and 20-mm spheres were so low owing to the partial volume effect, they were considered to be of limited use for optimisation and were thus excluded from the analysis.

**Fig. 2:**
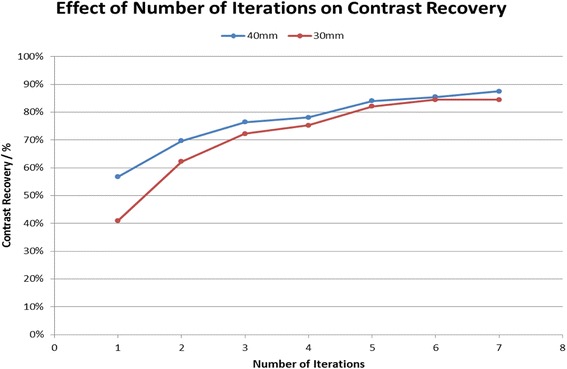
Number of iterations plotted against contrast recovery for the 40- and 30-mm sphere sizes. The number of subsets was 15

Figure 2 demonstrates that the CR increases with increasing iterations. The increase is initially quite marked, with the contrast of the 30-mm sphere rising from 41% (one iteration) to 72% (three iterations). Once five iterations are reached, however, it is clear that there is little gain in CR with increasing iterations. Furthermore, beyond this point, there is an increase in BV (30-mm sphere: six iterations BV = 6.3%, seven iterations BV = 8.9%).

Considering the results obtained in this analysis, it was decided that the optimised OSEM reconstruction for ^90^Y was 5 iterations and 15 subsets.

**Fig. 3:**
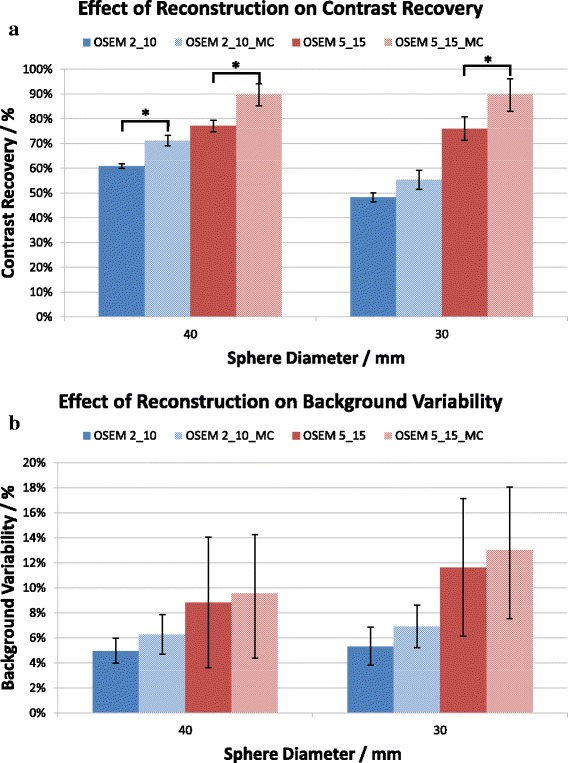
Effect of full Monte Carlo collimator modelling on **a** contrast recovery (*p* values left to right = 0.041, 0.048, 0.086, 0.040) and **b** background variability for the 40- and 30-mm sphere sizes. Error bars indicate the standard error

Following the optimisation of OSEM reconstruction, an investigation was performed into the effect of the full MC collimator modelling on each of the default and optimised OSEM reconstructions. Figure 3a demonstrates that the full MC collimator modelling improves the CR for default and optimised reconstructions, for both the 30- and 40-mm spheres. Student’s *t* tests were performed and identified that the improvement was statistically significant (*p* < 0.05) for all cases except the default reconstruction for the 30-mm sphere.

Though it seems that there is an increase in BV associated with the use of full MC collimator modelling, this difference is not statistically significant owing to the large variability in the values.

Quantitatively, it was concluded that the optimised OSEM reconstruction with full MC collimator modelling was the best reconstruction owing to the significant increases in CR observed. The clinical evaluation was then used to confirm whether the image quality of this reconstruction was also superior or whether the increase in background variability had led to a deterioration in image quality.

### Clinical evaluation

Results of the radiologist’s scoring were collated and each reconstruction was given a mark out of 20 by adding together the score in each of the four categories given in Table 1. Full results are given in Table 2.

**Table 2:** Results from clinical evaluation of patient images. Numbers are mean averages over all 10 patients and numbers in brackets indicate the standard deviation

Recon.	Overall image Quality		Lesion Detectability		Visibility of necrotic regions (where applicable)		Liver background Noise level		Total	
	No MC	MC	No MC	MC	No MC	MC	No MC	MC	No MC	MC
Default	3.5 (0.71)	3.5 (0.71)	3.4 (0.52)	3.2 (0.63)	3.2 (0.79)	3.3 (0.67)	5 (0)	5 (0)	15.1 (1.52)	15 (1.63)
Optimised	5 (0)	5 (0)	5 (0)	5 (0)	5 (0)	5 (0)	4.6 (0.52)	5 (0)	19.6 (0.52)	20 (0)

In every one of the 10 cases, the optimised OSEM reconstruction scored higher than the default OSEM reconstruction. Although the noise levels in the optimised OSEM images were higher, they were still considered to be acceptable by the radiologist and the increased contrast levels in the optimised OSEM resulted in it being scored more highly than the default reconstruction.

A Wilcoxon signed-ranks test was performed to test whether there was a statistically significant difference in each of the metrics described in Table 1 when full MC collimator modelling was used, compared to when it was not. The test revealed no significant differences for any of the metrics.

**Fig. 4:**
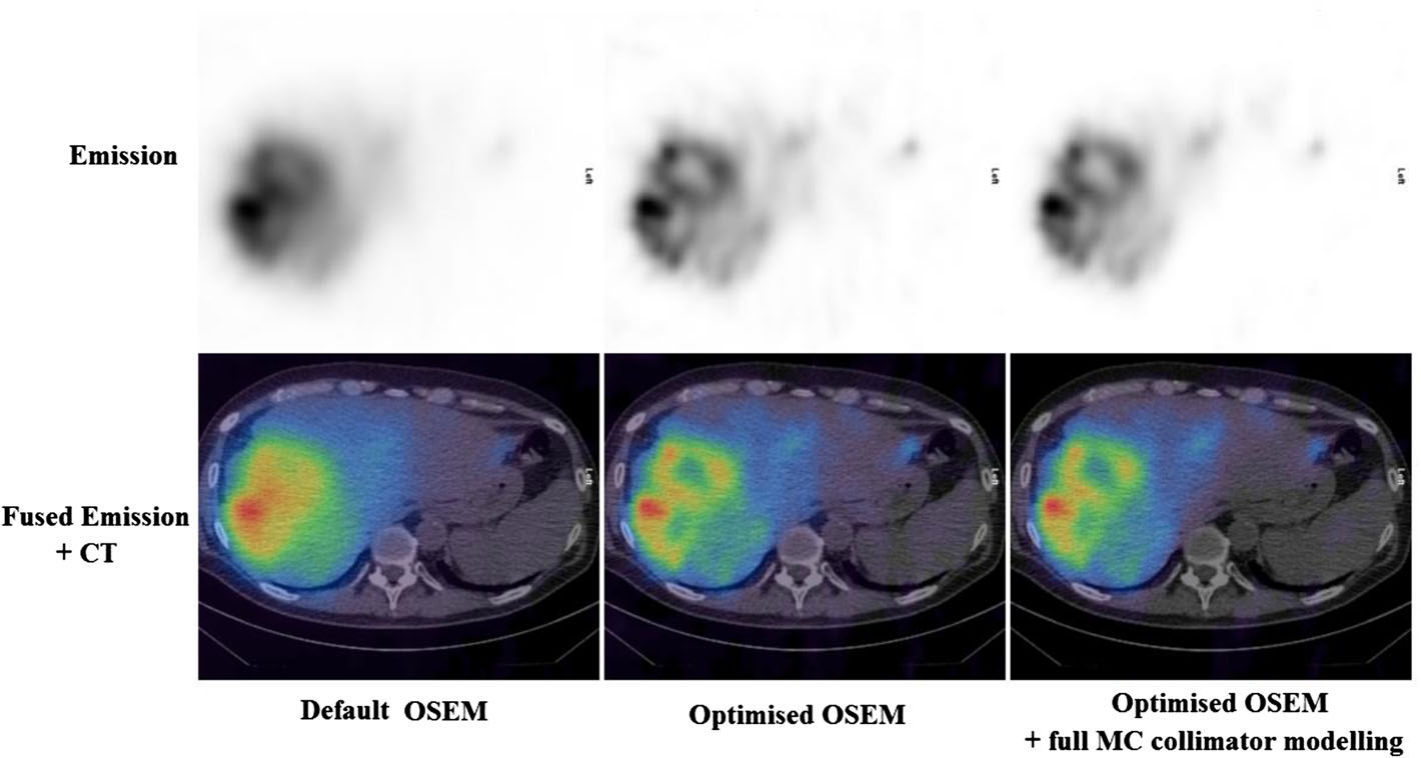
Transaxial slices showing distribution of microspheres in the liver of a patient. Left: default OSEM (2 iterations, 10 subsets); middle: optimised OSEM (5 iterations, 15 subsets); right: optimised OSEM with full MC collimator modelling

Figure 4 shows transaxial slices from one patient treated with ^90^Y microspheres, demonstrating the improvement in image quality gained through optimising the OSEM reconstruction protocol. Figure 4 also demonstrates that the image quality of the optimised OSEM reconstruction without full MC collimator modelling is visually similar to that of the optimised OSEM reconstruction with full MC collimator modelling.

## Discussion

The aim of this investigation into post-SIRT ^90^Y SPECT imaging was to optimise OSEM reconstruction and to assess whether full MC collimator modelling could be used to improve image quality and quantification. Based on these results, a recommendation of a single optimal reconstruction would be made.

The optimised OSEM protocol with full MC collimator modelling (5 iterations, 15 subsets) yielded a higher CR than the default OSEM protocol (2 iterations, 10 subsets), with an increase of over 42% (*p* < 0.001) observed for the 30 mm sphere. Even without the full MC collimator modelling, the optimised OSEM still demonstrated a great advantage in CR over the default protocol, with a 28% increase observed for the same sphere (*p* < 0.01). As well as performing best in the phantom optimisation, optimised OSEM also scored higher than the default OSEM protocol for all of the 10 patient datasets presented to the radiologist.

For both the optimised and default OSEM, the use of MC collimator modelling increased the CR values and, in a number of cases, this difference was significant (e.g., optimised OSEM CR increase = 14%, *p* = 0.048, 30-mm sphere). A previous study using the “Utrecht Monte Carlo System” has shown, using one acquisition of the NEMA IQ phantom, that Monte Carlo-based scatter correction for ^90^Y yielded a CR of 88% for a 37-mm sphere [14]. This study, using commercially available software, demonstrated a CR of 90% for a 30-mm sphere. A major limitation of ^90^Y SPECT imaging is the difficulty in applying traditional scatter correction techniques [5], which model the scatter within the object, owing to the continuous nature of the Bremsstrahlung spectrum and absence of a photopeak [14]. It is therefore not surprising that HybridRecon, which additionally models scatter within the detector and collimator, improves quantification of ^90^Y images.

It was found that BV does not differ significantly when MC collimator modelling is applied. Though from Fig. 3b it would appear that the BV does increase, the variability of the BV values between different acquisitions is so great that this increase is likely to be of no consequence (and it is not significant, *p* > 0.05), as confirmed by the results of the clinical study.

Both the phantom and patient dataset optimisation revealed the optimised OSEM reconstructions to be preferable to the default OSEM reconstructions. Having demonstrated that full MC collimator modelling improves CR, and thus quantification, without altering clinical image quality, it is therefore recommended that SPECT images of post-SIRT ^90^Y are reconstructed with this algorithm. For presentation to the radiologist for reporting, consideration should be given to applying a post-filter, but no post-filter should be applied when using the image for quantification purposes.

## Conclusions

OSEM with five iterations and 15 subsets produces significantly improved ^90^Y SPECT/CT images compared with the default protocol (2 iterations and 10 subsets). Use of full MC collimator modelling significantly improves CR, whilst not degrading image quality, making it suitable for aiding future quantification. Therefore, OSEM with five iterations and 15 subsets, incorporating full MC collimator modelling if available, is the recommended reconstruction algorithm for post-SIRT ^90^Y SPECT/CT imaging.
